# More Maternal Vascular Malperfusion and Chorioamnionitis in Placentas After Expectant Management vs. Immediate Delivery in Fetal Growth Restriction at (Near) Term: A Further Analysis of the DIGITAT Trial

**DOI:** 10.3389/fendo.2019.00238

**Published:** 2019-04-24

**Authors:** Marjon E. Feenstra, Mirthe H. Schoots, Torsten Plösch, Jelmer R. Prins, Sicco A. Scherjon, Albertus Timmer, Harry van Goor, Sanne J. Gordijn

**Affiliations:** ^1^Department of Obstetrics and Gynecology, University Medical Center Groningen, University of Groningen, Groningen, Netherlands; ^2^Department of Pathology and Medical Biology, University Medical Center Groningen, University of Groningen, Groningen, Netherlands

**Keywords:** fetal growth restriction, placental pathology, DIGITAT trial, maternal vascular malperfusion, chorioamnionitis

## Abstract

**Objective:** Management of late fetal growth restriction (FGR) is limited to adequate fetal monitoring and optimal timing of delivery. The *Disproportionate Intrauterine Growth Intervention Trial At Term* (DIGITAT) trial compared induction of labor with expectant management in pregnancies at (near) term complicated by suspected FGR. Findings of the DIGITAT trial were that expectant monitoring prolonged pregnancy for 10 days and increased birth weight with only 130 grams. This resulted in more infants born below the 2.3rd percentile compared to induction of labor, respectively, 12.5% in induction of labor and 30.6% in expectant monitoring group. The main placental lesions associated with FGR are maternal vascular malperfusion, fetal vascular malperfusion, and villitis of unknown etiology. We investigated whether placentas of pregnancies complicated with FGR in the expectant monitoring group reveal more and more severe pathology due to pregnancy prolongation.

**Material and methods:** The DIGITAT trial was a multicenter, randomized controlled trial with suspected FGR beyond 36 + 0 weeks. We now analyzed all available cases (*n* = 191) for placental pathology. The macroscopic details were collected and histological slides were recorded and classified by a single perinatal pathologist, blinded for pregnancy details and outcome. The different placental lesions were scored based on the latest international criteria for placental lesions as defined in the Amsterdam Placental Workshop Group Consensus Statement.

**Results:** The presence of maternal vascular malperfusion and chorioamnionitis were higher in the expectant management group (*p* < 0.05 and *p* < 0.01, respectively). No differences in placental weight and maturation of the placenta between the induction of labor and the expectant management group were seen. Fetal vascular malperfusion, villitis of unknown etiology and nucleated red blood cell count did not differ between the groups.

**Conclusion:** Expectant management of late FGR is associated with increased maternal vascular malperfusion and chorioamnionitis. This may have implications for fetal and neonatal outcome, such as programming in the developing child influencing health outcomes later in life.

## Introduction

Fetal growth restriction (FGR) is a condition in pregnancy in which the fetus fails to reach its growth potential. FGR affects up to 15% of all pregnancies (1, 2) and is not only associated with mortality, but also with long term morbidity (3–5). Appropriate placental nutrient and oxygen supply is essential for normal fetal growth. The origin of placental insufficiency is multifactorial (6, 7). Several types of lesions can be found in placentas of pregnancies complicated by FGR (7–9). The main placental lesions found in FGR placentas are maternal vascular malperfusion, fetal vascular malperfusion, and villitis of unknown etiology (9). Elevated nucleated red blood cells are considered as an indication of fetal hypoxia (10, 11). Other rare findings in placentas of FGR complicated pregnancies include chronic histiocytic intervillositis and massive perivillous fibrinoid deposition (12, 13).

Until now, adequate fetal monitoring and optimal timing of delivery are the only possible interventions for FGR as other therapeutic options are lacking (14). Timing of delivery in case of FGR is balanced between (relative) prematurity on the one side and prolonged undernutrition/hypoxia on the other side. The DIGITAT study investigated whether timely delivery in (near) term FGR would result in better outcomes. The primary analysis (36 + 0 till 41 + 0 weeks of gestation) showed equivalence in composite neonatal outcome between expectant monitoring and induction of labor (15, 16). Sub analysis revealed that the optimal time to minimize neonatal consequences is delivery around 38 weeks of gestation, when benefits of a planned delivery outweigh consequences of prolongation of pregnancy, as this is associated with a longer exposure to a malnourished environment (17). The DIGITAT trial found that expectant monitoring resulted in a pregnancy prolongation of a further 10 days compared to those in the immediate induction group. This was associated with an increase of only 130 grams in the birth weight of those expectantly managed and a higher proportion of infants born below the 2.3 percentile (30.6% compared to 12.5% in the immediate induction of labor group).

In this study we investigate whether placentas of pregnancies complicated with FGR at (near) term show specific features due to prolongation of pregnancy. The aim of this study is to assess the structural impact on the placenta when pregnancy is prolonged in FGR at term.

## Materials and Methods

### DIGITAT-Original Study Design

The *Disproportionate Intrauterine Growth Intervention Trial At Term* (DIGITAT) trial was a multicenter, randomized controlled trial of women pregnant with a singleton fetus with suspected FGR from 36 + 0 till 41 + 0 weeks of gestation onwards. Details of the DIGITAT trial protocol have been described elsewhere (15). In brief, consenting and eligible women were randomized to either expectant monitoring or induction of labor. The expectant monitoring group was monitored until the onset of spontaneous delivery or when for other reasons than suspected FGR an indication for delivery became apparent. In the induction of labor group delivery was induced within 48 h after randomization (17, 18).

### Data Collection of Further Analysis (Current Study)

All participating hospitals in the DIGITAT trial were asked to evaluate whether the placentas of their trial participants had been examined by a local pathologist. If so, all available material such as pathology reports, histologic slides, and tissue blocks were collected and re-analyzed. The analyses included details of the macroscopic description of the placental disk, membranes and umbilical cord as described in the placental pathology report.

Furthermore, the histologic slides, routinely stained with Hematoxylin and Eosin (H&E) were revised, scored and classified to latest international criteria (9) by a single perinatal pathologist (MS), who was blinded for pregnancy details and outcome.

### Placental Histology

The various placental lesions were evaluated based on the latest international criteria for placental lesions as described in the Amsterdam Placental Workshop Group Consensus Statement (9). Lesions evaluated were placental pathology consistent with maternal vascular malperfusion (MVM), fetal vascular malperfusion (FVM), chorioamnionitis (CA), villitis of unknown etiology (VUE), elevated nucleated red blood cell count (NRBC), chronic histiocytic intervillositis (CHI), and massive perivillous fibrin deposition (MPVFD). Also, if available, placental weight, placental weight percentile, birth weight placenta weight ratio (BWPW) (19), umbilical cord length and coiling index were included in the analyzes. The coiling index was calculated by dividing the number of whole coils by the umbilical cord length in centimeters. A normal coiling index is considered to be between 0.1 and 0.3. Due to the retrospective nature of this analysis unfortunately no other placental characteristic data (like the extension of infarction) were available.

### Statistical Analysis

SPSS 24.0 software for Windows (SPSS Inc. Chicago, IL) was used for the statistical analyses. Continued variables were evaluated with a two-tailed unpaired *T*-test and binary or categorical variables were evaluated with a Fisher Exact Test. Statistical significance was assumed at *p* < 0.05.

## Results

A total of 191 cases were available for review of placental pathology, respectively, 97 (321 in the original DIGITAT trail) participants in the induction of labor group and 94 (329 in original DIGITAT trail) participants in the expectant monitoring group.

In contrast to the original DIGITAT study the baseline characteristics of the two groups were statistically different for parity, maternal smoking and BMI (Table 1). Similar to the original DIGITAT study gestational hypertension was significantly higher in the expectant monitoring group. In the DIGITAT trial pre-eclampsia was more prominent in the expectant monitoring group than in the induction of labor group, but in our cohort of this re-analysis no significant difference was seen in the presence of pre-eclampsia.

**Table 1:** Demographic and baseline characteristics of the selected randomized participants for this analysis of placental pathology in FGR at term.

**Baseline characteristics**	**Induction of labor (*n* = 97)**	**Expectant monitoring (*n* = 94)**	**Sig**.
Nulliparous (%)	48	37	*p* = 0.013
Maternal age (years)	28.07 ± 5.58	27.65 ± 5.41	*p* = 0.500
BMI at study entry (kg/m^2^)	24.31 ± 6.09	23.18 ± 4.82	*p* = 0.024
Maternal smoking	58	39	*p* = 0.010
Gestational hypertension	7	13	*p* = 0.035
Pre-eclampsia	6	9	*p* = 0.150
**INCLUSION CRITERIA**			
Fetal abdominal circumference <10th percentile	76	82	*p* = 0.211
Estimated fetal weight <10th percentile	95	76	*p* = 0.224
Deceleration of fetal abdominal circumference curve	14	26	*p* = 0.038
**MODE OF DELIVERY**			
Induced labor	95	51	*p* = 0.001
Vaginal delivery	43	39	*p* = 0.100
Vaginal instrumental	33	34	*p* = 0.352
Cesarean section	24	27	*p* = 0.248
**NEONATAL CHARACTERISTICS**			
Gestational age at birth (days)	264 ± 7.71	272 ± 9.27	*p* = 0.010
Birth weight (grams)	2242.23 ± 285	2303.10 ± 365	*p* = 0.065
Length of neonatal stay in hospital (days)	8.9 ± 7.00	9.1 ± 8.41	*p* = 0.690
Admission to the neonatal intensive care unit (NICU)	2	5	*p* = 0.020

*Data is presented as proportion, unless stated otherwise in the table. Significance is presented as p-value, a p < 0.05 is assumed significant*.

Most women in the current analysis met the DIGITAT study criteria of FGR by combinations of inclusion criteria, usually by a fetal abdominal circumference (AC) below the 10th percentile (respectively *n* = 74 in induction of labor and *n* = 77 in expectant monitoring) and/or an estimated fetal weight (EFW) below the 10th percentile (respectively *n* = 92 in the induction of labor and *n* = 72 in the expectant monitoring). Furthermore, a decline in AC growth was also a criterium for study inclusion (not as a single criterium, but in combination with either AC or EFW below p10) and was differently observed between the groups, with a higher presence in the expectant monitoring group (induction of labor was *n* = 14 and expectant monitoring *n* = 24; *p* < 0.05) (Table 1).

In the expectant monitoring group in 51 of the 94 cases labor was induced due to either maternal indication (8), fetal indication (20), combined maternal and fetal indication (4), or rupture of membranes (2) (Table 1).

In the current analysis, women in the expectant monitoring group had a longer gestational age (8 days), compared to the induction of labor group (*p* < 0.01). This is slightly less prolongation than in the original DIGITAT study in which pregnancy was prolonged by 10 days. Birth weight in the study population of this sub analysis was not significantly different between the groups. However, there was a difference of 60 grams between the expectant management and induction of labor, which means a weight gain of 60 grams in the achieved 8 days of pregnancy prolongation. This is also slightly less than in the original DIGITAT study (with 130 grams weight gain in 10 days) (Table 1). The average neonatal stay in hospital was approximately 8 days in both groups. A few cases needed admission to the neonatal intensive care unit, significantly more in the expectant monitoring group (*p* < 0.05). The length of stay in the neonatal intensive care unit was approximately 6 days for both groups. There were no neonatal deaths reported in both groups.

### Placental Characteristics

In Table 2 placental characteristics are described of both the induction of labor group and the expectant monitoring group. No differences in placental weight, umbilical cord length, coiling index and BWPW ratio were found between the two groups.

**Table 2:** Comparison of placental characteristics between the induction of labor and expectant monitoring group in FGR at term.

**Placental characteristics**	**Induction of labor (*n* = 97)**	**Expectant monitoring (*n* = 94)**	**Sig**.
Placental weight (grams)	371.49 ± 15.50	361.40 ± 15.54	*p* = 0.707
Umbilical cord length (cm)	35.44 ± 1.92	35.36 ± 1.93	*p* = 0.789
Coiling index	0.35 ± 0.189	0.30 ± 0.216	*p* = 0.483
Birth Weight to Placental Weight ratio (BWPW)	6.29 ± 1.336	6.71 ± 1.787	*p* = 0.284

*Data is presented as mean. Significance is presented as p-value, a p < 0.05 is assumed significant. No statistical differences were found in placental characteristics*.

### Placental Histology

The findings of placental histology in both groups are described in Table 3. Maturation of the placenta was similar in both groups. Maternal vascular malperfusion (MVM) was significantly higher in the expectant monitoring group compared to the induction of labor group (*p* < 0.05). Fetal vascular malperfusion (FVM) was similar in both groups. In placentas from the expectant monitoring group we found a significantly higher incidence of chorioamnionitis (CA) (*p* < 0.01). The presence of villitis of unknown etiology (VUE) and the nucleated red blood cell count (NRBC) was similar in both groups. Rare placental findings like chronic histiocytic intervillositis (CHI) and massive perivillous fibrinoid deposition (MPFD) were seen in a few cases (*n* = 2 for CHI in the induction group and 0 in the expectant monitoring group; *n* = 4 and *n* = 1 for MPFD in the induction group and expectant monitoring group, respectively).

**Table 3:** The comparison of placental pathology between induction of labor and expectant monitoring in FGR at term.

**Placental histology**	**Induction of labor (*n* = 97)**	**Expectant monitoring (*n* = 94)**	**Sig**.
Maturation matching gestational age (MAT)	89	82	*p* = 0.701
Maternal vascular malperfusion (MVM)	9	17	*p* = 0.049
Fetal vascular malperfusion (FVM)	10	16	*p* = 0.069
Chorioamnionitis (CA)	13	32	*p* = 0.010
Villitis of unknown etiology (VUE)	29	30	*p* = 0.507
Elevated nucleated Red Blood Cell Count (NRBC)	2	3	*p* = 0.485
Chronic histiocytic intervillositis (CHI)	2	0	*p* = 0.257
Massive perivillous fibrinoid deposition (MPFD)	4	1	*p* = 0.194

*Data is presented as proportion in the table. Significance is presented as p-value, a p < 0.05 is considered significant*.

## Discussion

In this sub analysis of the DIGITAT study we found that maternal vascular malperfusion (MVM) occurred significantly more often in the expectant monitoring group in comparison to the induction of labor group (*p* < 0.05) in (near) term FGR. Moreover, participants in the expectant monitoring group had a significantly higher incidence of chorioamnionitis (CA) (*p* < 0.01). The DIGITAT trial previously showed no differences in composite neonatal outcome between expectant monitoring and induction of labor, however, it was seen that the average weight gain was only 130 grams in 10 days delivery delay, which resulted in more babies born with a birth weight below the p2.3. The follow up study of the original DIGITAT trial at 2 years of age showed that babies born with a birth weight below p2.3 scored lower at the ages and stages questionnaire (ASQ) for developmental disorders.

MVM occurs early in pregnancy and is likely due to maladaptation of the spiral arteries. It develops over time when gestation prolongs (21, 22). By inadequate remodeling of spiral arteries a high resistance flow induces shear stress and damage to parenchyma, like infarctions, and differences in oxygen tension leading to oxidative stress and free radical damage (12, 22–25). The higher prevalence of MVM in the expectant monitoring group could be caused by a prolonged time of hypoxia caused by inadequate maternal vascularization. This could further lead to increase in cellular stress, e.g., oxidative stress and endoplasmic reticulum (ER) stress. This may ultimately lead to an increase in the occurrence of FGR and development of preeclampsia (23–26).

To support this theory of prolonged exposure to a pathologic environment, a significant higher prevalence of the development of gestational hypertension in the expectant monitoring group was seen. MVM has been described to be causal in gestational hypertension and preeclampsia (20, 27). This finding further strengthens the clinical experience that expectant monitoring or prolongation of pregnancy in FGR at (near) term is not without risks especially for the mother (28), even though on short term no differences were seen in neonatal outcome. A study from Parra-Saavedra et al. implies in a group of late onset Small-for-Gestational Age (SGA) that placental under perfusion (PUP) can also lead to higher neonatal morbidity; in this study in 77/84 placentas maternal vascular supply was—as in our study- compromised (29). Furthermore, similar to the original study, in this sub-study a large group of women had labor induced although being allocated to the expectant monitoring group (51/94: 66%), indicating that the difference between the groups in MVM is in fact an underestimation of what would really happen when delivery was awaited.

The histologic diagnosis of chorioamnionitis (CA) is made when an inflammatory infiltrate is seen in the chorioamniotic membranes. At term CA is most frequently induced by inflammation and cellular stress, in the absence of a microorganism (30). During labor the uteroplacental blood flow is compromised leading to hypoxia of the placenta and its membranes. This leads to an increase in pro-inflammatory markers in the placenta. In fact, labor at term has been associated with infiltration of inflammatory cells in the placenta and higher plasma levels of pro-inflammatory chemokines and cytokines, such as Interleukin 6 (IL-6), Interleukin 8 (IL-8), and Tumor Necrosis Factor alpha (TNF-alpha) (30–32). Hypothetically, the inflammatory response in the chorioamniotic membranes and cervix could be the result of an increase in pro-inflammatory cytokines caused by cellular stress, e.g., oxidative stress and ER stress (23, 24, 33, 34).

Furthermore, even though in the expectant monitoring group labor was often induced, significantly more spontaneous deliveries took place in the expectant monitoring group. Recent studies showed a higher incidence of histological chorioamnionitis in spontaneous labor compared to, respectively, induced labor, or cesarean section (33, 35). This could be an additional explanation for the significantly higher incidence of chorioamnionitis in the expectant monitoring group.

In our analysis, we observed a higher prevalence of CA in the expectant monitoring group. As described above, this is conceivably explained by increase of pro-inflammatory cytokines due to the prolonged hypoxic environment.

The incidence of fetal vascular malperfusion (FVM) was not significantly different although a higher number of cases were seen in the expectant monitoring group in comparison with induction of labor.

Like MVM, FVM has been associated with fetal growth restriction (FGR) due to decreased availability of functional parenchyma (36). The etiology of FVM is however multifactorial and not specifically related to FGR. FVM has been associated with multiple factors as placental (for example umbilical cord pathology), maternal (for example preeclampsia), and fetal (for example cardiac malformations) etiologies (6, 37, 38). Also, intra-amniotic infection with a fetal response (vasculitis) and chronic villitis of unknown etiology (VUE) are known associations (39).

In our study, villitis of unknown etiology (VUE) occurred in approximately 30% of all cases, distributed equally between both randomized groups. In existing literature, a broad range of incidences of VUE is described, partly because the definition has not been uniform and also due to differences in sampling of placental parenchyma (40–43). With an equal distribution between our groups, we conclude that prolonged pregnancy in the expectant monitoring group is not associated with an increased prevalence of VUE.

At term, normally only a small number of circulating nucleated red blood cells (NRBCs) are seen in the fetal circulation, if any (11). In response to fetal hypoxia the NRBC count increases (10, 11). In our study, only a few cases showed elevated NRBCs, suggesting that fetal monitoring to prevent fetal hypoxia has been adequate in the two randomized groups as no differences were seen in number of cases with elevated NRBCs.

In addition to the placental lesions described above, we also investigated the presence of chronic histiocytic intervillositis (CHI), massive perivillous fibrinoid deposition (MPFD), and maternal floor infarction (MFI). These are rare placental lesions, associated with (severe) FGR, with high recurrence risks (12, 13, 44–47). In our analyses, we found 2 cases of CHI in the induction of labor group and none in the expectant monitoring group. Five cases of MPFD were encountered, four of which in the induction of labor group and one in the expectant monitoring group. No cases with maternal floor infarction (MFI) were found in the total study population. No significant differences were seen between the induction of labor group and the expectant monitoring group for CHI and MPFD.

We did not find any differences in placental weight or birth weight to placental weight ratio (BWPW ratio) (Table 2). It has been described that placental weight from fetuses with FGR is significantly lower than placentas from fetuses with a normal birth weight and that the BWPW ratio is higher, although in fact fetal weight per placental unit is high and the placenta performed well in that sense. A low BWPW ration (a relatively low fetal weight per placental unit) has also been described in relation to adverse outcome (19). Given the fact that pregnancy prolongation resulted in only little weight gain and placental weights were not different the BWPW ratio was, as expected lower (although not significant) in the induction group (8, 48, 49).

## Conclusion

Placental pathology in FGR encompasses a broad group of diagnoses, not exclusive for FGR. Our study shows that expectant monitoring is associated with a higher incidence of maternal vascular malperfusion (MVM) and chorioamnionitis (CA), whilst fetal vascular malperfusion, severe acute fetal hypoxia and villitis of unknown etiology are similar in both groups. This study further strengthens the results from a previous sub-analysis that prolongation of gestation has no additional benefits and even increases the prevalence of MVM and CA in FGR; both placental findings are associated with an increase in children born with severe growth restriction and are possibly associated with unfavorable outcome at later ages.

### Strengths and Limitations of the Study

The strengths of this study are a dedicated, uniform examination of the placentas, done by one perinatal pathologist unaware of clinical details, by the latest criteria, in a large number of placentas from pregnancies complicated with FGR. In this sub-study prolongation of pregnancy is 2 days less than the original DIGITAT study and the weight gain in that period is on average less per day (13 grams per day in the original study vs. 7.5 grams in this study). This combined with the fact that more inclusions showed a decline in AC growth than the original study and finally that more inductions in the expectant management group of this analysis (66%) occurred than in the original study (25.5%) indicates that there is probably an inclusion bias with the more severe cases included in the study. We however believe that this inclusion bias does not undermine the results of this study. In the induction group there may also be a similar trend in sending more placenta's of clinically severe cases to the pathologist than the clinically less severe cases. A limitation of this study is that only 29% cases of the original DIGITAT trial were included, due to the availability of placental histological samples, which were collected from all participating hospitals in the Netherlands. We believe it is of great importance to analyze the placenta histopathology for understanding the underlying pathophysiology, especially in cases of FGR in which a common mechanism is placental insufficiency. All in all, we found that expectant management of late FGR is associated with increased maternal vascular malperfusion and chorioamnionitis. Further studies on the clinical implications of these findings are warranted.

## Ethics Statement

This study was carried out in accordance with the recommendations of DIGITAT Guidelines, Leiden University Medical Centre. The protocol was approved by the METC LUMC P04.210.

## Author Contributions

MF, MS, JP, TP, SS, AT, HvG, and SG contributed to the design and implementation of the research, to the analysis of the results, and to the writing of the manuscript.

### Conflict of Interest Statement

The authors declare that the research was conducted in the absence of any commercial or financial relationships that could be construed as a potential conflict of interest.
